# Early Clinical Approach Prevents Severe Neurotoxicity Following Cobra Envenoming: An Integrated Experimental and Multi-Center Clinical Study in Thailand

**DOI:** 10.3390/biomedicines14010144

**Published:** 2026-01-10

**Authors:** Sethapong Lertsakulbunlue, Musleeha Chesor, Panuwat Promsorn, Wanida Chuaikhongthong, Wipapan Khimmaktong, Wittawat Chantkran, Janeyuth Chaisakul

**Affiliations:** 1 Department of Pharmacology, Phramongkutklao College of Medicine, Bangkok 10400, Thailand; sethapong.ler@pcm.ac.th; 2 Faculty of Medicine, Princess of Naradhiwas University, Narathiwat 96000, Thailand; musleeha.c@pnu.ac.th; 3 Galyani Vadhana Karun Hospital, Faculty of Medicine, Princess of Naradhiwas University, Narathiwat 96000, Thailand; panuwat.p@pnu.ac.th; 4 Division of Health and Applied Sciences, Faculty of Science, Prince of Songkla University, Songkhla 90110, Thailand; chuaikhongthong@gmail.com (W.C.); wipapan.k@psu.ac.th (W.K.); 5 Department of Pathology, Phramongkutklao College of Medicine, Bangkok 10400, Thailand; chantkran@yahoo.com

**Keywords:** snakebite, paralysis, rats, ptosis, antivenom, patients

## Abstract

**Background:** Cobras (*Naja* sp.) are medically important snakes in Thailand. Envenoming by the monocled cobra (*N. kaouthia*) often causes neurotoxicity, most notably ptosis, ophthalmoplegia, local tissue necrosis and progressive paralysis leading to respiratory failure. Early antivenom administration and respiratory support are medically significant for effective treatment. **Methods**: In this study, we determined the association between the time course of cobra envenoming and related neurotoxic outcomes using the clinical profiles of cobra envenomed patients. We also demonstrated histopathological changes in the neuromuscular junction of the diaphragm in experimentally envenomed rats. **Results**: A retrospective study of 69 cases of cobra envenoming in Central and Southern Thailand shows that delayed arrival beyond one hour at hospital was common among younger adults (47.0% aged 10–29) and associated with more severe neurotoxicity, including higher rates of ptosis (41.2%, *p* = 0.032) and referrals (41.2% vs. 15.4%, *p* = 0.040). Antivenoms (22 Monovalent and 1 Polyvalent) were administered to 23 (33.3%) envenomed victims and caused adverse reactions in 9 cases (39.1%). Neurotoxicity following cobra envenoming in the clinical section correlated with histopathological examination of envenomed rat diaphragms. Transmission electron microscopy (TEM) revealed degeneration of the neuromuscular junction and diaphragm within 1 h following experimental cobra envenomation, which worsened by 4 h. Intravenous administration of antivenom at recommended doses reduced diaphragmatic damage but failed to prevent presynaptic degeneration after 90 min of envenoming. **Conclusions:** Clinically, extraocular muscle paralysis was the earliest manifestation. Early monitoring and prompt administration of antivenom are essential to reduce neurotoxicity and relevant complications.

## 1. Introduction

Snakebite envenoming is a critically neglected tropical disease that disproportionately affects impoverished, rural communities in low- and middle-income countries, particularly in Sub-Saharan Africa, South America, and South to Southeast Asia, where access to medical infrastructure remains limited [1,2,3]. Each year, venomous snakebites affect an estimated 5.8 million people worldwide, resulting in 81,000 to 138,000 deaths, according to the World Health Organization (WHO). Southeast Asia alone accounts for up to 70% of these fatalities [4]. Thailand’s lush tropical environment harbors a diverse range of snake species, including both venomous and non-venomous varieties, leading to frequent encounters between humans and snakes. Nationally, snakebites affect approximately 8525 to 8906 individuals each year, causing 2 to 7 deaths and up to 7 amputations [5].

Cobras are medically significant elapids in snakebite-related mortality, not only in Thailand but across South and Southeast Asia [6,7,8]. In Thailand, cobras, including *Naja kaouthia* (monocled cobra), *Naja sumatrana* (equatorial spitting cobra), *Naja siamensis* (Indochinese spitting cobra), *Naja fuxi*, and *Ophiophagus hannah* (king cobra), release venom that disrupts neuromuscular transmission, leading to muscle weakness, ptosis, dysarthria, dysphagia, and potentially fatal respiratory paralysis [7]. Moreover, among the cobras, spitting cobras, particularly *N. siamensis* and *N. sumatrana* pose a unique clinical challenge due to their ability to project venom toward the eyes of perceived threats [6,9,10]. This behavior can cause venom ophthalmia, which may lead to chemical conjunctivitis, corneal erosion, or secondary infection if not promptly irrigated. Although immediate flushing often prevents severe complications, some patients may still develop delayed ocular symptoms, underscoring the need for early recognition and appropriate first-aid interventions [11].

Prompt and appropriate treatment is crucial in reducing mortality and morbidity associated with cobra envenoming. The early administration of specific immunoglobulin G (IgG) antibodies, known as snake antivenom, remains the cornerstone of effective management. Two types of antivenom are available: monovalent, targeting a specific snake species, and polyvalent, which covers multiple species and is particularly valuable when the biting species cannot be identified [12]. Evidence underscores the effectiveness of early intervention in cobra envenomed cases in a series involving 20 cobra-bitten Thai farmers, only one fatality occurred, with the remaining patients surviving after receiving antivenom [13]. Similarly, a retrospective review of 91 neurotoxic snakebite cases in Bangkok identified two deaths from cobra envenomation, while none occurred among hematotoxic snakebite cases, highlighting the need for rapid diagnosis and antivenom delivery in neurotoxic bites [14].

Cobra envenomation poses significant clinical challenges due to the complexity of its toxic components, venom pharmacokinetics, and diverse clinical effects. Understanding the composition of cobra venom, such as three-finger toxins (3FTX), to which postsynaptic neurotoxins and cardiotoxins (cytotoxins: CTXs) belong [15] is necessary in order to improve antivenom efficacy and patient outcomes. Moreover, prior studies have demonstrated substantial variability in venom absorption and distribution, with intramuscular bioavailability ranging from 4% to 81.5%, reflecting marked interspecies and interindividual differences [16]. Administration of specific antivenom, including endotracheal intubation with ventilator support, is required to prevent fatality from neurotoxic outcomes. Despite timely antivenom administration, certain venom components, particularly CTXs and 3FTXs, may persist in local tissues such as bullae, wound exudates, and necrotic lesions [15]. This persistence highlights the limitations of systemic antivenom in neutralizing tissue-bound venom and suggests a role for adjunctive measures such as surgical debridement [17].

However, important gaps remain in our understanding of early outcomes after cobra envenoming, particularly within the first hour. Furthermore, comprehensive clinical characterizations of cobra envenomation cases are limited, constraining the development of targeted therapeutic strategies [18]. This study aims to address these gaps by (1) investigating the association between neurotoxicity observed following cobra envenoming and morphological changes in the neuromuscular junction using an animal model, and (2) analyzing the clinical characteristics of envenomated patients using multi-center data from hospitals in southern and central Thailand, where cobra bites are endemic [6,13]. This dual approach aims to enhance our understanding of cobra envenomation and facilitate the development of more effective treatment protocols.

## 2. Materials and Methods

### 2.1. Study Design and Subjects

Snakebite data were retrospectively obtained from Pattananikom Hospital, a 60-bed community hospital in Lopburi, Central Thailand. The hospital provides primary care in a district with a high incidence of fatal snakebite. Secondary data were abstracted from patients presenting to the emergency department between 1 October 2014 and 30 August 2023 [19,20,21,22]. In addition, a retrospective descriptive study of cobra envenomation was conducted using records from five hospitals in Narathiwat, Thailand’s southernmost province, covering 1 November 2016 to 31 October 2021. These hospitals were Ra-Ngae Hospital (120 beds), Rueso Hospital (90 beds), Yi-Ngo Hospital 80th Anniversary Commemoration Hospital (60 beds), Takbai Hospital (120 beds), and Naradhiwas Rajanagarindra Hospital (400 beds), which functions as the provincial referral center [23].

### 2.2. Human Ethical Considerations

The Institutional Review Board of the Royal Thai Army Medical Department conforms to international guidelines such as the Declaration of Helsinki, the Belmont Report, Council for International Organizations of Medical Sciences Guidelines, and the International Conference on Harmonization of Technical Requirements for Registration of Pharmaceuticals for Human Use-Good Clinical Practice (ICH-GCP), which reviewed and approved the study (Approval no. S097h/64_Exp and S029h/66_Exp). Given that the study used secondary data, the requirement for informed consent documentation was waived, and the Institutional Review Board of the Royal Thai Army Medical Department authorized the waiver.

### 2.3. Data Collection

The authors extracted data from patient medical records using a standardized case record form, which included three main components: (1) baseline demographic and clinical information at the emergency department (ED), such as age, gender, date of visit, time of arrival at the ED, time of envenomation, bite location, snake species, and any prehospital interventions; (2) laboratory findings obtained at the ED prior to treatment initiation, including complete blood count (CBC), serum electrolyte levels, serum creatinine (Cr), 20 min whole blood clotting time (20WBCT), venous clotting time (VCT), and international normalized ratio (INR), along with subsequent 20WBCT and VCT values; and (3) treatment and outcome data, including presenting symptoms, admission details, antivenom administration, and other medications given during the visit. Snakebite cases were identified using the International Classification of Diseases 10th Revision (ICD-10) code T63.0, as noted in the patient records [24], and further reviewed to confirm cobra envenomation based on physician documentation. Records coded as T63.0 were excluded if the physician’s diagnosis indicated a different type or unknown type of snake.

According to guidance from the Queen Saovabha Memorial Institute (QSMI), indications for neurotoxic antivenom therapy include signs of muscle weakness, ptosis, and confirmed bites from either the banded krait or Malayan krait. The recommended dose ranges from 5 to 10 vials, typically administered over 30 to 60 min via intravenous infusion [18]. Laboratory reference ranges include sodium levels of 135–145 mEq/L, potassium levels of 3.6–5.1 mEq/L, and bicarbonate levels between 22 and 29 mEq/L [25]. Serum creatinine of above 1.2 mg/dL were considered [26].

### 2.4. Histopathological Study Using a Rat Model

#### 2.4.1. Snake Venom

*N. kaouthia* (monocled cobra) venom was given by Professor Nison Sattayasai (Department of Biochemistry, Faculty of Science, Khon Kaen University, Thailand). The venom sample was obtained by milking multiple specimens held captive at the Queen Saovabha Memorial Institute (QSMI) of the Thai Red Cross Society in Bangkok, Thailand. Freeze-dried venom samples were pooled and stored at 4 °C prior to use. When required, the venom was weighed and reconstituted in phosphate-buffered saline (PBS), and the protein concentration was determined using a BCA protein assay (Pierce Biotechnology, Rockford, IL, USA).

#### 2.4.2. Antivenoms

The Neuro-polyvalent antivenom (NPAV; Lot no. NP00117; expiry date: 4 August 2022) was purchased from QSMI. The freeze-dried antivenoms were dissolved with pharmaceutical-grade water supplied by the manufacturer. The dissolved antivenoms were then stored at 4 °C prior to use.

#### 2.4.3. Animal Ethics and Care

All procedures were performed in accordance with the relevant guidelines and regulations (https://arriveguidelines.org, accessed on 10 March 2024). In brief, male Sprague–Dawley rats were purchased from Nomura-Siam International Co. Ltd., Bangkok, Thailand. All animals were maintained on a regular diurnal lighting cycle (12:12 light–dark). Two rats were housed together in individual stainless-steel cages with ad libitum access to food and drinking water. Chopped corn cobs were used as bedding. Approval for all experimental procedures was obtained from the Subcommittee for Multidisciplinary Laboratory and Animal Usage of Phramongkutklao College of Medicine and the Institutional Review Board, Royal Thai Army Department, Bangkok, Thailand (documentary proof of ethical clearance number: IRBRTA S029b/67_Xmp on 9 April 2024) in accordance with the 1986 U.K. Animal (Scientific Procedure) Act and the National Institutes of Health’s Guide for the Care and Use of Laboratory Animals (NIH Publications 8th edition, 2011).

#### 2.4.4. Anesthetized Rat Preparation for Monitoring Blood Pressure and Heart Rate Following the Administration of Cobra Venom

Male Sprague–Dawley rats (280–350 g: *n* = 3–5 per group) were anesthetized using separate intraperitoneal injections of Zoletil (20 mg/kg, Virbac, TX, USA) and Xylazine (5 mg/kg, Kepro, Woerden, The Netherlands). Additional anesthetic was administered as required throughout the experiment. A midline incision was made in the cervical region, and cannulae were inserted into the trachea, jugular vein and carotid artery for artificial respiration (if required), the administration of antivenom and the measurement of blood pressure, respectively. Arterial blood pressure was recorded using a MLT0380/D pressure transducer (ADInstruments, Bella Vista, Australia) filled with heparinised saline (25 U/mL). Systemic blood pressure was monitored using a MacLab system (ADInstruments, Bella Vista, Australia). The rats were kept under a heat lamp for the entire experiment to maintain body temperature. *N. kaouthia* venom (2 mg/kg) was intramuscularly (i.m.) administered through the right gastrocnemius muscle. Additionally, the venom dose was selected in a preliminary experiment as mentioned in the previous study [27]. In brief, the effect of *N. kaouthia* venom was studied in a preliminary test by i.m. administration of doses of 0.5, 1.0, and 2.0 mg/kg to rats (*n* = 3 per dose). Venom doses of 0.5 and 1.0 mg/kg (i.m.) failed to cause morphological change in the rat’s diaphragm. Therefore, a dose of 2 mg/kg (i.m.) was subsequently chosen for further experiments.

Then, rats’ diaphragms were collected for histopathological determination under the Transmission Electron Microscopy [27].

#### 2.4.5. Transmission Electron Microscopy (TEM)

Rat diaphragms were cut into small pieces (~1 mm^3^) and immediately fixed in 2.5% buffered glutaraldehyde. The tissues were post-fixed in 1% osmium tetroxide, dehydrated, infiltrated with propylene oxide and embedded in resin. Semi-thin sections of approximately 0.5 µm or 1.0 µm were stained with Toluidine blue, used as a guideline to the area of interest and further trimmed. Ultrathin sections of about 60 nm were cut on an ultramicrotome and then stained with uranyl acetate and lead citrate. The ultrathin sections were mostly spread on 200- or 300-mesh copper grids and stained with uranyl acetate and lead citrate. The sections were then studied and photographed for morphological changes using a transmission electron microscope (TEM, JEM-2010, JEOL, Ltd., Tokyo, Japan).

#### 2.4.6. Data Analysis

Statistical analysis was done using R version 4.4.1. Data normality was assessed using the Kolmogorov–Smirnov test. Descriptive statistics were applied to summarize baseline characteristics. Continuous variables were reported as means with standard deviations (SD) or medians with interquartile ranges (IQR), depending on data distribution. Categorical variables were summarized using frequencies and percentages. To compare continuous variables, independent *t*-tests were used for normally distributed data, while the Mann–Whitney U-test was applied for non-normally distributed data. Comparisons between categorical variables were conducted using Pearson’s chi-squared or Fisher’s exact test, where applicable. A two-tailed *p*-value of less than 0.05 was considered statistically significant. Moreover, to assess the association between systemic envenomation and patient characteristics, cases of venom ophthalmia were excluded, as they are unlikely to result in systemic effects [28,29,30].

## 3. Results

### 3.1. Baseline Characteristics of Participants

A total of 83 participants were included in the study. Table 1 compares participant characteristics across hospital regions, as snake fauna may differ by region. Of these, 24 cases (28.9%) were from the central region, and 59 cases (71.1%) were from the southern region. Overall, the clinical characteristics of cobra envenomation cases were comparable between the two regions (*p* > 0.05). However, all 10 reported cases of venom ophthalmia occurred in the central region.

### 3.2. Association Between Patient Characteristics, Laboratory Findings, and Bite-to-Hospital Time Within 1 h

**Figure 1:**
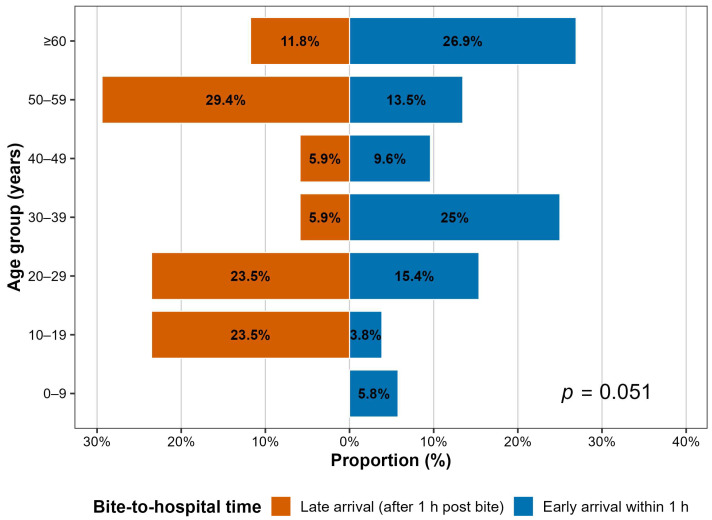
Age distribution of participants stratified by bite-to-hospital time within 1 h.

Table 2 shows the patient characteristics and laboratory findings stratified by bite-to-hospital time within 1 h. Ten cases were diagnosed with venom ophthalmia and four cases with missing bite-to-hospital time were excluded, yielding a final sample of 69 cases for analysis. Among these, 17 patients (24.6%) presented to the hospital more than one hour after being bitten. Overall, the median age of participants was 42 years (IQR: 27–55), with no significant difference between groups. However, when stratified by bite-to-hospital time within 1 h, the age distribution appeared bimodal, with a predominance in both working-age and older adults. Notably, participants aged 10–19 years were more likely to present after 1 h (*p* = 0.051) (Figure 1). Ten cases (71.4%) bitten during the morning hours arrived at the hospital more than 1 h after the bite (*p* = 0.092). Regarding clinical signs and symptoms, ptosis was observed in 7 cases (41.2%) who arrived after 1 h (*p* = 0.032), while muscle weakness was reported in 2 cases (11.8%) within the same group (*p* = 0.148). Furthermore, the proportion of patients with metabolic acidosis was higher among those who arrived more than 1 h after the bite (33.3%), although this difference did not reach statistical significance (*p* = 0.082).

### 3.3. Association Between Clinical Management and Bite-to-Hospital Time Within 1 h

Table 3 summarizes the clinical management of patients stratified by time from bite to hospital arrival within 1 h. Regarding prehospital management, a twisting tourniquet was applied in 9 of 69 patients (13.3%). Among those who arrived more than 1 h after the bite, 7 cases (41.2%) were referred to other facilities, compared to only 8 cases (15.4%) among those who arrived within 1 h (*p* = 0.040). The administration of medications, including NSAIDs, paracetamol, tramadol, antibiotics, corticosteroids, and tetanus toxoid, was comparable between the two groups. However, intubation was performed in 5 patients (29.4%) who arrived after 1 h, versus 7 patients (13.5%) who presented earlier (*p* = 0.152).

Table 4 presents details of antivenom administration stratified by bite-to-hospital time within 1 h. A total of 23 patients received antivenom. Although not statistically significant (*p* = 0.167), a higher proportion of patients who arrived more than 1 h after the bite received antivenom (8 cases, 47.1%) compared to those who arrived within 1 h (15 cases, 28.8%). Among patients who received antivenom and arrived early, the majority (92.9%) were administered 10 vials. In contrast, among those who arrived after 1 h, 1 patient (12.5%) received 3 vials and 3 patients (37.5%) received 5 vials at the emergency department. Furthermore, nine participants reported adverse events: six reported an itchy rash, two reported nausea, and one reported chest tightness.

### 3.4. Histopathological Examination of Neuromuscular Junction in Rat Diaphragm Following Intramuscular Administration of Naja kaouthia Venom and the Effectiveness of Neuro-Polyvalent Antivenom

**Figure 2:**
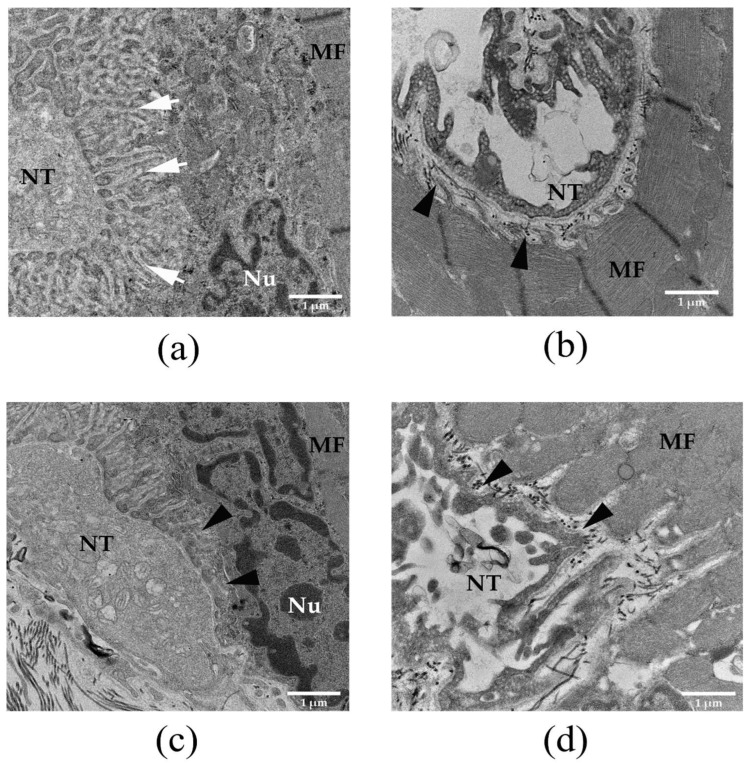
Morphological changes in the rat neuromuscular junction (TEM: scale bar = 1 µm) after the i.m. administration of (**a**) saline (200 µL) showing nerve terminal (NT), muscle fiber (MF), nucleus (Nu) and synaptic fold (white arrow). The administration of *N. kaouthia* venom (2 mg/kg: i.m.) for (**b**) 60 and (**c**) 90 min caused disorganized synaptic folds and partial loss of synaptic folds (black arrowheads). (**d**) TEM image of rat diaphragm shows severe degeneration and loss of the synaptic fold (black arrowheads) following i.m. administration of *N. kaouthia* venom (2 mg/kg) for 4 h (*n* = 4).

**Figure 3:**
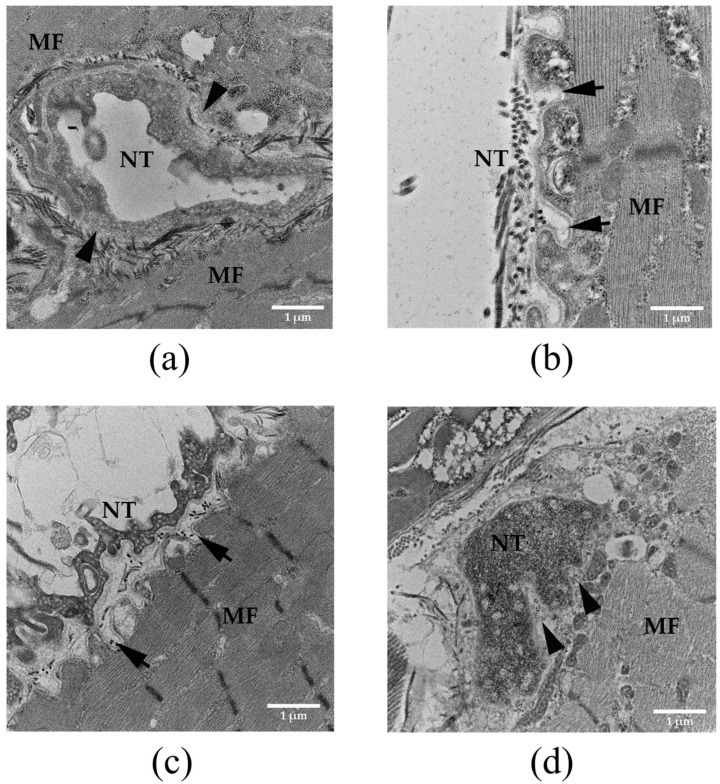
Morphological changes in the rat neuromuscular junction (TEM: scale bar = 1 µm) after the i.m. administration of (**a**) *N. kaouthia* venom (2 mg/kg: i.m.) for 4 h, showing severe degeneration and loss of the synaptic fold (black arrowheads). The appearance of the synaptic fold (black arrows) in the rat diaphragm (TEM analysis: scale bar = 2 µm) after i.v. administration of NPAV at (**b**) 30 and (**c**) 60 min following the administration of *N. kaouthia* (2 mg/kg, i.m.). (**d**) Administration of NPAV at 90 min following the administration of *N. kaouthia* (2 mg/kg, i.m.) failed to prevent the severe degeneration of the synaptic fold (black arrowheads) (*n* = 4).

The administration of saline did not cause a marked histopathological change in the neuromuscular junction of the diaphragm (Figure 2a). The administration of *N. kaouthia* venom (2 mg/kg: i.m.) for 60 and 90 min (Figure 2b and Figure 2c, respectively) caused the degeneration and loss of synaptic folds. The severe degeneration of synaptic fold was detected following the i.m. administration of *N. kaouthia* venom for 4 h (Figure 2d and Figure 3a). The intravenous (i.v.) administration of neuro-polyvalent antivenom (NPAV) at 30 and 60 min prevented the degeneration of the synaptic fold following the administration of *N. kaouthia* venom (2 mg/kg, i.m.; Figure 3b and Figure 3c, respectively). The i.v. administration of NPAV at 90 min failed to prevent presynaptic degeneration (Figure 3d) following the administration of *N. kaouthia* venom (2 mg/kg: i.m. *n* = 4).

**Figure 4:**
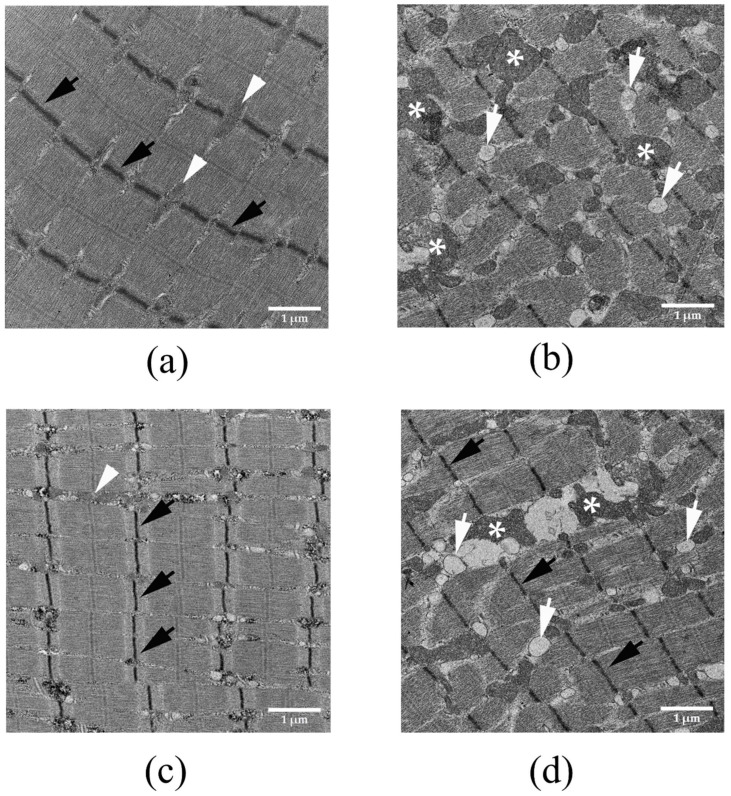
Morphological changes in the rat diaphragm (TEM: scale bar = 1 µm) after the i.m. administration of (**a**) saline showing Z band (black arrows) and mitochondria (white arrowhead), (**b**) The i.m. administration of *N. kaouthia* venom (2 mg/kg) for 4 h causes the swelling and degeneration of mitochondria (white asterisks) and intramyocellular lipid droplets (white arrows). (**c**) The appearance of the Z band (black arrows) and normal mitochondria (white arrowhead) in the rat diaphragm after i.v. administration of NPAV at 30 min following the administration of *N. kaouthia* (2 mg/kg, i.m.). (**d**) The i.v. administration of NPAV at 90 min following the administration of *N. kaouthia* (2 mg/kg, i.m.) did not prevent the swelling and degeneration of mitochondria (white asterisks) and intramyocellular lipid droplets (white arrows).

The administration of saline did not cause a marked histopathological change in muscle fibre of the diaphragm (Figure 4a). The severe degenerations of diaphragmatic muscle were detected following the i.m. administration of *N. kaouthia* venom for 4 h (Figure 4b). The i.v. administration of NPAV at 30 min prevented the degeneration of the muscle fibre of the diaphragm following the i.m. administration of *N. kaouthia* venom (Figure 4c). The administration of NPAV (i.v.) at 90 min following the i.m. administration of *N. kaouthia* venom failed to prevent mitochondrial swelling of muscle fibre (Figure 4d).

## 4. Discussion

Snakebite envenoming is a neglected tropical disease that causes significant mortality and disability. Early first aid, rapid assessment, and timely antivenom can mean the difference between life and death. This study presents concurrent findings from both an animal model and retrospective data on cobra envenomation, demonstrating morphological changes in the rat diaphragm 60 min after venom administration, which correspond to the onset of neurological signs in patients who arrived more than one hour after the bite. The characteristics of patients who arrived more than one hour after envenomation were also illustrated, particularly among younger adults.

Neurotoxicity and death due to respiratory failure and infection of the bite wound have been reported following envenoming by *N. kaouthia* in Thailand [7]. Neurotoxic symptoms, including bilateral ptosis, limb weakness, breathlessness, dysphonia and dysphagia, are clinically significant in the diagnosis and treatment of systemic cobra envenoming [3]. These envenomed outcomes have been reported to be due to the presence of phospholipase A_2_ (PLA_2_)_,_ Three-finger toxins (3FTxs) and postsynaptic neurotoxins, causing an inhibition of neurotransmission at the motor endplate [9].

Previous studies usually focused on the inhibitory effects of cobra venoms/toxins on neuromuscular transmission and tissue necrosis, specifically on the postsynaptic nicotinic acetylcholine receptors and cardiotoxins (CdTX) [6,7,15]. In the current study, intramuscular (i.m.) administration of 2 mg/kg of *N. kaouthia* venom induced morphological changes in the rat diaphragm and nerve terminals. The administration of *N. kaouthia* venom (2 mg/kg) also caused an undetectable heart rate and blood pressure in anesthetized rats following i.m. injection for 4 h. In fact, a *N. kaouthia* venom dose of 2 mg/kg (i.m.) is equivalent to 9xLD50 (9 times the dose required to kill half the tested mice following venom administration), according to a previous study by Leong et al. (2012) [8]. Additionally, variation in snake venom LD50 was found to be associated with the route of administration, including proteomic variations and the source of the venom [8].

Recently, we detected severe pathological changes in rat diaphragm and nerve terminals following i.m. administration of *N. kaouthia* venom at 2 mg/kg [27]. These morphological changes included skeletal muscle injury, a high degree of synaptic degeneration, and loss of synapses, which were diminished by the early administration of cobra monovalent antivenom (i.e., 30 min after experimentally envenoming) [27]. In the current study, histopathological changes in the diaphragm following i.m. administration of cobra venom were initially detected at 60 min, with mild injury to the synaptic membrane. A higher degree of synaptic degeneration was detected following experimental envenoming at 90 min and 4 h. This indicates that the severity of synaptic degeneration was time-dependent and induced by the cytotoxic peptides in cobra venoms such as PLA_2_ and Cytotoxins (CTXs) [9,15].

Clinically, a retrospective study reported a median interval of 1 h (range: 10 min to 24 h) between the cobra bite and the onset of neurological symptoms [7], consistent with our histopathological findings. Moreover, our retrospective clinical analysis shows that among those who presented to the emergency department (ER) more than 1 h late, ptosis was significantly greater.

Approximately 24.6% of patients in this study presented to the emergency room (ER) more than one hour after envenomation, with delayed arrival particularly common among younger adults aged 10–29 years. A study in rural Sri Lanka reported a similar proportion (14%) of patients who intentionally delayed treatment by a median of 45 min (IQR 20–120), often waiting for symptoms to appear [31]. Similarly, a qualitative study in Brazil found that delayed patient transport was associated with snakebites sustained during daily work activities and with personal perceptions of severity [32]. In our study, young adults who were bitten during the day were more likely to present to the hospital late (71.4% in the morning, 45.8% in the afternoon) compared with those bitten at night. Moreover, a related study also reported that younger age groups were more likely to exhibit greater severity in neurotoxic outcomes than those over 30 years, possibly due to higher exposure risk, lower body mass, and faster venom absorption [33]. These findings emphasize the importance of raising awareness of snakebite among young workers and promoting prompt treatment-seeking behavior following snakebite envenomation.

The administration of snake antivenom is a mainstay of treatment for cobra envenoming. The indication for antivenom administration includes any clinical signs and symptoms of skeletal muscle paralysis or weakness. The Queen Saovabha Memorial Institute (Thai Red Cross Society, Bangkok, Thailand) is the only manufacturer of cobra antivenom (*N. kaouthia* antivenom; NKAV) in Thailand. It also produces the Neuro Polyvalent Snake Antivenom (NPAV) for Southeast Asian elapid envenoming, which covers the venoms of King cobra (*Ophiophagus hannah*), monocled cobra (*N. kaouthia*), banded krait (*Bungarus fasciatus*) and Malayan krait (*Bungarus candidus*) [8]. Our previous study demonstrated that early administration of *N. kaouthia* monovalent antivenom (NKAV) prevented morphological changes in rat diaphragm following experimental cobra envenoming [27]. In the present study, we determined the effectiveness of neuro-polyvalent antivenom (NPAV) to prevent cobra venom-induced degeneration of the rat diaphragm. The histopathological changes in the rat diaphragm following cobra envenoming were minimized by the intravenous administration of NPAV at the recommended therapeutic dose (i.e., 1 mL antivenom per 0.6 mg of *N. kaouthia* venom). However, the therapeutic effect of snake antivenom depends on the time of administration following snakebite envenoming. Delayed antivenom administration at 90 min failed to prevent synaptic degeneration following cobra envenoming. In addition, our study demonstrated the efficacy of expired antivenom in neutralizing morphological changes in the diaphragm and nerve terminals of envenomed animals. This confirmed the stability of expired snake antivenom from QSMI of the Thai Red Cross Society. Our data provide hope in the cases of snakebite emergencies when preferred antivenoms are unavailable [34,35].

In our clinical study, antivenom for cobra envenomation was administered; however, patients who presented later tended to receive fewer vials. The higher referral rate may explain this among late presenters, which serves as a proxy for more severe cases and potentially reduces the likelihood that they received antivenom at our facility. Given the high proportion of unidentified snakebite cases, accurate species identification is crucial for ensuring appropriate antivenom administration, particularly because not all hospitals stock polyvalent snake antivenom [22]. Early administration of antivenom is essential to neutralize snake toxins before irreversible damage occurs. Delayed administration of snake antivenom may be difficult to prevent or reverse flaccid paralysis, coagulopathy, acute kidney injury, and tissue necrosis [12,36,37]. In addition, supportive treatments such as pain management, immobilization, and wound care should be initiated promptly to minimize further complications.

The strength of our current study lies in its status as one of the few to demonstrate a correlation between experimental studies using animal models and the clinical characteristics of cobra envenomation in patients. These data indicate that late arrival at the hospital, or delayed treatment longer than one hour following a snakebite, is associated with severe neurotoxicity and related symptoms. Our clinical data are also consistent with histopathological studies in experimentally envenomed rats, which show that late administration of snake antivenom beyond 60 min is ineffective at reversing tissue damage.

Nevertheless, several limitations should be acknowledged. First, the cross-sectional design restricts the ability to assess longitudinal changes and long-term outcomes after discharge. Second, the retrospective nature of the study and its 10-year span may have led to missing data due to incomplete medical records. Hence, some information may be unavailable. For instance, the reason for the variation in the number of vials received per patient could not be determined. Third, reliance on the primary physician’s emergency department report for arrival times may introduce reporting bias. Moreover, further management during hospitalization was not collected. Fourth, tissue damage was examined indirectly through rat models and inferred for humans, as direct investigation in patients was not feasible; moreover, the 1 h post-envenomation window in rodents cannot be directly equated to 1 h in humans because of interspecies differences in body size, circulation time, and metabolic rate, so this extrapolation should be interpreted with caution. Fifth, the time to antivenom administration was not directly captured; thus, delays may have occurred even among patients arriving early, although this is likely minimal since all cases were treated in the emergency department and involved cobra envenomation [31,33]. Finally, although multicenter, the study included only 69 cases, which may limit statistical power. Despite this, consistent trends in outcomes were observed, but caution is warranted when generalizing the findings.

## 5. Conclusions

This study demonstrates histopathological changes in experimentally envenomed animals and poorer clinical outcomes when cobra envenomation extends beyond one hour, underscoring the critical importance of timely antivenom treatment. We also observed the effectiveness of expired neuro-polyvalent antivenom in preventing cobra venom-induced tissue injury, which might be an option for snake envenoming treatment when antivenom is in short supply or unavailable, thereby compromising public health. Extraocular muscle paralysis was the earliest clinical manifestation of cobra envenomation in this cohort. Patients presenting more than one hour after the bite tended to have worse outcomes, underscoring the need for early monitoring and prompt administration of appropriate antivenom to reduce neurotoxicity and related complications. Despite the limitations of a small retrospective study, these findings support efforts to strengthen public awareness and ensure rapid access to care for snakebite in Thailand.

## Figures and Tables

**Table 1:** Characteristics of participants stratified by hospital region.

Characteristic	Central	Southern	*p*-Value ^2^
	N = 24 ^1^	N = 59 ^1^	
Year of visit			0.216
2012 to 2015	2 (8.3%)	N/A	
2016 to 2019	10 (41.7%)	34 (57.6%)	
2020 to 2023	12 (50.0%)	25 (42.4%)	
Visited months			0.094
Jan to Mar	4 (16.7%)	14 (23.7%)	
Apr to Jun	9 (37.5%)	11 (18.6%)	
Jul to Sep	4 (16.7%)	23 (39.0%)	
Oct to Dec	7 (29.2%)	11 (18.6%)	
Gender			0.734
Male	16 (66.7%)	37 (62.7%)	
Female	8 (33.3%)	22 (37.3%)	
Age (years)	43 (25, 54)	37 (27, 61)	0.864
Age group (years)			0.296
0–9	0 (0.0%)	3 (5.1%)	
10–19	5 (20.8%)	5 (8.5%)	
20–29	2 (8.3%)	10 (16.9%)	
30–39	4 (16.7%)	12 (20.3%)	
40–49	5 (20.8%)	6 (10.2%)	
50–59	5 (20.8%)	8 (13.6%)	
≥60	3 (12.5%)	15 (25.4%)	
Admit	17 (70.8%)	52 (88.1%)	0.102
Refer ^3^	6 (25.0%)	11 (18.6%)	0.555
Bite Site			<0.001
Arm	2 (8.3%)	8 (13.6%)	
Chest	0 (0.0%)	1 (1.7%)	
Eye	10 (41.7%)	0 (0.0%)	
Foot	9 (37.5%)	30 (50.8%)	
Hand	1 (4.2%)	9 (15.3%)	
Leg	0 (0.0%)	10 (16.9%)	
Not seen	2 (8.3%)	1 (1.7%)	
Time before arrival			0.551
<30 min	8 (47.1%)	18 (31.0%)	
30 to 60 min	6 (35.3%)	26 (44.8%)	
1 to 4 h	2 (11.8%)	5 (8.6%)	
4 to 24 h	1 (5.9%)	9 (15.5%)	
more than 1 day	0 (0.0%)	0 (0.0%)	
Bitten in the morning	8 (53.3%)	28 (52.8%)	0.973
Signs and Symptoms			
Muscle weakness	2 (8.3%)	1 (1.7%)	0.199
Dyspnea	1 (4.2%)	5 (8.5%)	0.667
Bruising	2 (8.3%)	8 (13.6%)	0.716
Necrotic	1 (4.2%)	3 (5.1%)	>0.999
Ptosis	2 (8.3%)	12 (20.3%)	0.331
Asymptomatic	0 (0.0%)	6 (10.2%)	0.175
Endotracheal intubation	2 (8.3%)	10 (16.9%)	0.494
Prehospital managements			
Wash wounds with water	0 (0.0%)	2 (3.4%)	>0.999
Twisting tourniquet	0 (0.0%)	9 (15.3%)	0.053
Folk doctor	0 (0.0%)	0 (0.0%)	>0.999
Sodium (mEq/L)			>0.999
Hyponatremia (<135)	1 (4.8%)	2 (4.3%)	
Normal (135–145)	20 (95.2%)	43 (91.5%)	
Hypernatremia (>145)	0 (0.0%)	2 (4.3%)	
Potassium (mEq/L)			0.772
Hypokalemia (<3.5)	5 (23.8%)	14 (29.8%)	
Normal (3.5–5.5)	16 (76.2%)	33 (70.2%)	
Hyperkalemia (>5.5)	0 (0.0%)	0 (0.0%)	
Chloride (mEq/L)			0.186
Hypochloremia (<90)	0 (0.0%)	0 (0.0%)	
Normal (90–105)	14 (66.7%)	21 (46.7%)	
Hyperchloramia (>105)	7 (33.3%)	24 (53.3%)	
Bicarbonate (mEq/L)			0.705
Metabolic acidosis (<20)	2 (9.5%)	6 (14.6%)	
No metabolic acidosis (≥20)	19 (90.5%)	35 (85.4%)	
Serum creatinine (mg/dL)			0.681
Normal	20 (87.0%)	41 (91.1%)	
Elevated	3 (13.0%)	4 (8.9%)	

^1^ *n* (%); Median (Q1, Q3). ^2^ Pearson’s Chi-squared test; Fisher’s exact test; Wilcoxon rank sum test. ^3^ Some cases were referred after being admitted.

**Table 2:** Characteristics and laboratory examinations of participants stratified by time to hospital, excluding venom ophthalmia.

Characteristic	Total	Early Arrival Within 1 h	Late Arrival (After 1 h Post Bite)	*p*-Value ^2^
	N = 69	N = 52 ^1^	N= 17 ^1^	
Visited months				0.394
Jan to Mar	16 (21.9%)	13 (25.0%)	3 (17.6%)	
Apr to Jun	18 (24.7%)	9 (17.3%)	6 (35.3%)	
Jul to Sep	25 (34.2%)	20 (38.5%)	4 (23.5%)	
Oct to Dec	14 (19.2%)	10 (19.2%)	4 (23.5%)	
Gender				0.500
Male	46 (63.0%)	32 (61.5%)	12 (70.6%)	
Female	27 (37.0%)	20 (38.5%)	5 (29.4%)	
Age (years)	42 (27, 55)	41 (30, 62)	36 (21, 50)	0.256
Age group (years)				0.051
0–9	3 (4.1%)	3 (5.8%)	0 (0.0%)	
10–19	7 (9.6%)	2 (3.8%)	4 (23.5%)	
20–29	12 (16.4%)	8 (15.4%)	4 (23.5%)	
30–39	14 (19.2%)	13 (25.0%)	1 (5.9%)	
40–49	9 (12.3%)	5 (9.6%)	1 (5.9%)	
50–59	12 (16.4%)	7 (13.5%)	5 (29.4%)	
≥60	16 (21.9%)	14 (26.9%)	2 (11.8%)	
Bite site				0.550
Arm	10 (13.7%)	6 (11.5%)	3 (17.6%)	
Chest	1 (1.4%)	0 (0.0%)	1 (5.9%)	
Foot	39 (53.4%)	28 (53.8%)	8 (47.1%)	
Hand	10 (13.7%)	7 (13.5%)	3 (17.6%)	
Leg	10 (13.7%)	8 (15.4%)	2 (11.8%)	
Not seen	3 (4.1%)	3 (5.8%)	0 (0.0%)	
Bitten in the morning	33 (52.4%)	22 (45.8%)	10 (71.4%)	0.092
Signs and Symptoms				
Muscle weakness	3 (4.1%)	1 (1.9%)	2 (11.8%)	0.148
Dyspnea	6 (8.2%)	4 (7.7%)	2 (11.8%)	0.631
Bruising	10 (13.7%)	9 (17.3%)	0 (0.0%)	0.100
Necrotic	4 (5.5%)	2 (3.8%)	1 (5.9%)	>0.999
Ptosis	14 (19.2%)	7 (13.5%)	7 (41.2%)	0.032
Asymptomatic	6 (8.2%)	6 (11.5%)	0 (0.0%)	0.324
Sodium (mEq/L)				>0.999
Hyponatremia (<135)	3 (5.0%)	2 (4.7%)	0 (0.0%)	
Normal (135–145)	55 (91.7%)	39 (90.7%)	13 (100.0%)	
Hypernatremia (>145)	2 (3.3%)	2 (4.7%)	0 (0.0%)	
Potassium (mEq/L)				0.477
Hypokalemia (<3.5)	16 (26.7%)	13 (30.2%)	2 (15.4%)	
Normal (3.5–5.5)	44 (73.3%)	30 (69.8%)	11 (84.6%)	
Hyperkalemia (>5.5)	0 (0.0%)	0 (0.0%)	0 (0.0%)	
Chloride (mEq/L)				0.202
Hypochloremia (<90)	0 (0.0%)	0 (0.0%)	0 (0.0%)	
Normal (90–105)	30 (51.7%)	23 (56.1%)	4 (30.8%)	
Hyperchloramia (>105)	28 (48.3%)	18 (43.9%)	9 (69.2%)	
Bicarbonate (mEq/L)				0.082
Metabolic acidosis (<20)	8 (14.8%)	4 (10.5%)	4 (33.3%)	
No metabolic acidosis (≥20)	46 (85.2%)	34 (89.5%)	8 (66.7%)	
Serum creatinine (mg/dL)				0.320
Normal	53 (89.8%)	35 (85.4%)	14 (100.0%)	
Elevated	6 (10.2%)	6 (14.6%)	0 (0.0%)	

^1^ *n* (%); Median (Q1, Q3). ^2^ Fisher’s exact test; Pearson’s Chi-squared test; Wilcoxon rank sum test.

**Table 3:** Clinical management stratified by time before arrival.

Characteristic	Early Arrival Within 1 h	Late Arrival (After 1 h Post Bite)	*p*-Value ^2^
	N = 52 ^1^	N = 17 ^1^	
Prehospital management			
Wash wounds with water	1 (1.9%)	1 (5.9%)	0.435
Twisting tourniquet	9 (17.3%)	0 (0.0%)	0.100
Folk doctor	0 (0.0%)	0 (0.0%)	>0.999
Mouth removal of toxin	0 (0.0%)	0 (0.0%)	>0.999
Admit	42 (80.8%)	15 (88.2%)	0.716
Refer ^3^	8 (15.4%)	7 (41.2%)	0.040
Drug administration			
NSAIDs	3 (6.8%)	2 (14.3%)	0.585
Paracetamol	34 (77.3%)	10 (71.4%)	0.725
Tramadol	15 (34.1%)	2 (14.3%)	0.195
Antibiotics	40 (76.9%)	13 (76.5%)	>0.999
Corticosteroids	32 (61.5%)	11 (64.7%)	0.815
Tetanus Toxoid	14 (26.9%)	3 (17.6%)	0.533
Wound debridement	5 (9.6%)	0 (0.0%)	0.323
Endotracheal Intubation	7 (13.5%)	5 (29.4%)	0.152

^1^ *n* (%). ^2^ Fisher’s exact test; Pearson’s Chi-squared test. ^3^ Some cases were referred after being admitted.

**Table 4:** Antivenom administration stratified by time before arrival.

Characteristic	Early Arrival Within 1 h	Late Arrival (After 1 h Post Bite)	*p*-Value ^2^
	N = 52 ^1^	N = 17 ^1^	
Antivenom	15 (28.8%)	8 (47.1%)	0.167
Neuropolyvalent	1 (1.9%)	0 (0.0%)	>0.999
Cobra monovalent	14 (26.9%)	8 (47.1%)	0.122
Number of vials			0.017
3	0 (0.0%)	1 (12.5%)	
5	0 (0.0%)	3 (37.5%)	
10	13 (92.9%)	4 (50.0%)	
12	1 (7.1%)	0 (0.0%)	
Median (Q1, Q3)	10 (10, 10)	7.5 (5, 10)	0.007
Side effects	6 (40.0%)	3 (37.5%)	>0.999

^1^ *n* (%). ^2^ Pearson’s Chi-squared test; Fisher’s exact test; Mann–Whitney U test.

## Data Availability

The datasets generated during and/or analyzed during the current study are available from the corresponding author on reasonable request.

## Abbreviations

The following abbreviations are used in this manuscript:

AV	antivenom
ED	emergency department
NPAV	Neuro Polyvalent Snake Antivenom
TEM	Transmission Electron Microscopy
WBC	whole blood clotting time
VCT	venous clotting time
INR	international normalized ratio
Cr	creatinine
IQR	interquartile range
SD	standard deviation
i.m.	intramuscular/intramuscularly
